# What does it take to conduct clinical trials in African countries? Insights from Morocco

**DOI:** 10.3332/ecancer.2022.1411

**Published:** 2022-06-13

**Authors:** Nada Benhima, Mohamed El Fadli, Ismail Essâdi, Rhizlane Belbaraka

**Affiliations:** 1 Medical Oncology Department, Mohammed VI University Hospital, Marrakech 40080, Morocco; 2 Faculty of Medicine and Pharmacy, Cadi Ayyad University, Marrakech 40080, Morocco; 3 Medical Oncology Department, Avicenne Military Hospital, Marrakech, Morocco

**Keywords:** cancer, Morocco, North Africa, clinical trials, health equity

## Abstract

The progress on cancer diagnosis and treatment has attained, in the last decade, enormous achievements by any estimate. Immunotherapy, new generations of targeted therapies, Chimeric antigen T-cells, cancer vaccines and the fascinating breakthroughs in translational research and cancer biology have changed the direction of cancer care. However, the fact that all patients worldwide cannot have access to these advances is dramatic. Alongside this, taking part in clinical research is one way to improve and invest in cancer care. Patients from African—and most low-resources countries—are rarely offered the chance of being included in clinical trials.

This well-known fact paints a disheartening picture of what having cancer is like in the poorest settings. This situation will further decline with population aging, major changes in risk profile imported from developed countries and life expectancy increasing in most African countries. If no radical changes are made, this North–South contrast will become more critical and continue to grow. Yet, there is room for hope because only when we acknowledge the problem can we begin to address it. We need a better understanding of the reasons behind this gap and to advocate for more representation from African patients in clinical trials, with respect to the socio-economic, epidemiological and unique demands of each country across the continent.

## Background

African countries experience the greatest burden of cancer, with above-average mortality rates in comparison to developed countries. The predictions of the International Agency for Research on Cancer estimate that over the next 5 years new annual cancer cases will attain more than one million (1.30 M) in Africa and are expected to increase to 2.08 million by 2040 [1]. The scarcity of healthcare resources—estimated based on health expenditure per capita—and the limited access to advanced treatments add fuel to the flame of the burden of cancer in the continent.

Clinical research is a part of a long and careful process in which randomised clinical trials are the engines that drive progress in cancer treatment and improve the delivery of cancer care. In the poorest countries, where basic cancer research is much needed and most valuable, hosting clinical trials has not been smooth sailing. While countries with limited resources endure the highest burden of cancer, most practice-changing studies are being hosted in North America, Europe and Asia, attracting greater investments from the ever-growing and ever-evolving pharmaceutical industry. It is, however, insufficient to presume that their consequent results would be extrapolated to the worldwide population, regardless of the ethnic and the genetic diversities specific to each country, especially in an era of increasing emphasis on personalised medicine.

A 2018 article in PLOS medicine examined the trial density – all fields included – over 7 years. It revealed that from the year 2006 through 2012, 83% of the clinical trial sites operated in 25 high-income countries, and only 5% were conducted in 91 lower-middle or low-income countries (LMICs) [2]. This established fact of lower representation of medically underserved populations in oncology clinical trials continues to widen the health equity gap and the entire cancer care continuum to the detriment of the most disadvantaged populations of the Third World. Recently, a special issue of JCO Global Oncology [3] has been published to identify operational issues affecting oncology clinical trials in Africa as part of a large-scale project to develop an African Virtual Platform for Oncology Clinical Trials Registry. A quantitative web-based retrospective review of open cancer clinical trials in all African countries was conducted. The results confirmed the dearth and underrepresentation of Africans in oncology clinical trials, with the majority of studies being located in six countries. Egypt, South Africa and Algeria were ranked on the top of the list with, respectively, 45, 11 and 10 clinical trials open to inclusion by 2020.

To our great surprise, although Morocco shares common socio-economic, epidemiologic and healthcare particularities that are unique to the region of North Africa, the country was found to host only four open interventional clinical trials that are assessing a cancer-directed therapy mainly sponsored by pharmaceutical companies with marginal funding from academic institutions. For clarity, except for a few published phase II trials, there has been no local phase III trial conducted by Moroccan teams. Herein, we make an overall assessment of the ongoing phase III interventional clinical trials on cancer in Morocco to improve their visibility and point to the key issues that hinder the interest of pharmaceutical companies in conducting clinical trials in the country. We describe secondly the local strategies that have been adopted to leverage active participation in the clinical research process.

## Materials and methods

We conducted a web-based review of open clinical trials in Morocco. The review was guided by the above-cited JCO-GO special issue framework. The electronic databases included in the review are clinicaltrials.gov (http://clinialtrials.gov), Pan-African Clinical Trials Registry (http://www.pactr.org), CenterWatch (http://centerwatch.com), World Health Organisation International Clinical Trials Registry Platform (http://apps.who.int/trialsearch/Default.aspx), European Union Clinical Trials Register (https://www.clinicaltrialsregister.eu) and the Moroccan Ministry of Health website (https://www.sante.gov.ma). Online specific registries of sub-Saharan countries were omitted from the review. For greater details, the reader is referred to the special two-part series of editorial articles on clinical trials in Africa published by the ASCO Academic Global Oncology.

We secondly carried out a bibliographical search in PubMed, Medline, Web of Science and Scopus databases, using combinations of terms “clinical trials” AND “Cancer” AND “Morocco” AND/OR “North-Africa”. The websites of local cancer associations were also included in the search, namely AMFROM “Moroccan Association for Training and Research in Medical Oncology” and SMC “Moroccan Society of Cancer”. Informal reports from local physicians that are involved in clinical trials’ activities were obtained to complete the review.

## Results

### Current ongoing cancer clinical trials in North African countries

As we draft this paper, a total of three interventional multicentric phase III studies are open to recruitment (*n* = 1) or yet to be initiated (*n* = 2) in Morocco. Twelve studies have completed patient enrolment (*n* = 11) or ended prematurely before achieving their intended recruitment rate (*n* = 1). Trials that were active, but no longer recruiting at the time of analysis, were considered ‘completed’ as they had completed their patient recruitment.

Of the three found trials, the only one open to recruitment trial (Impassion132) is investigating the efficacy and safety of immunotherapy in early relapse, locally advanced or metastatic triple-negative breast cancer. The trial is active in 4 African countries (South Africa, Morocco, Algeria and Egypt) out of a total of 30 countries worldwide. The second trial sponsored by the same Swiss pharmaceutical company is investigating the combination of anti-TIGIT and anti-PD-L1 cancer immunotherapies on unresectable oesophageal squamous cell carcinoma. The trial is currently active in 220 sites spread over 28 countries. Morocco is the second African country expected to host the trial after South Africa.

These findings lead us to the conclusion that, so far, only three interventional phase III trials are available for nearly all cancer patients in the country. Regarding their eligibility criteria that are too restrictive, one cannot expect that the number of patients who may be offered the inclusion will be noticeable. Table 1 describes the ongoing interventional clinical trials in oncology open to recruitment in the Maghreb region (Morocco, Algeria and Tunisia).

## The case of Egypt

Over 100 clinical cancer drug trials are being currently conducted in Egypt, making it the first country for hosting cancer clinical trials on the continent. Most studies are assessing cancer-direct drugs, mostly phase III, followed by phase IV, real-world studies. The majority are conducted in Cairo and Alexandria. Industry-sponsored trials carried out the greatest share. Breast cancer is the most presented in trials volume, with the most frequent intervention being drug, followed by procedure.

## Discussion

### Barriers to hosting trials in Morocco

Although barriers to trial participation in Africa have lately been the subject of numerous papers, most have focused on recruitment barriers in the WHO African region, while North African countries received less attention. Also, most studies surveyed the patient’s perspective to a greater extent, while the standpoint of physicians was less explored.

The scoping review outlined, in a well-structured, detailed and documented framework, the multifaceted challenges of conducting clinical trials in the African continent. The sequels of limited human, financial and infrastructural facilities, worsened by the low prioritisation of research and the complexity of regulatory processes, are common barriers that increase the cost-effectiveness of clinical trials’ interventions and make it harder to undertake in African countries. Patient under-recruitment creates an additional blockade that continues to hinder the interest of research organisations and pharmaceutical companies.

At the most local level, Morocco’s near-absence from the North African clinical trials map is of utmost concern. Although there are substantial gaps in our understanding of the rationale behind this geographical disparity, several issues that may be specific to our country emerge.

#### Limited infrastructure facilities

In the absence of a comprehensive list of cancer treatment facilities in the region, comparisons cannot be fairly made. For clarity, the number of university-affiliated public oncology centres ranges from 6 in Morocco to 17 anti-cancer centres in Algeria and up to 26 in Egypt. The number of centres included does increase the recruitment rate, and trials that include high-volume cancer centres are more likely to reach recruitment targets within the expected timeframe [4]. In Morocco, medical oncology was recognised as a separate academic and clinical discipline in 1994. Over the past 20 years, 150 medical oncologists were formed and over 120 fellows are currently trained [5]. In parallel, the Algerian population is being served by almost 1000 certified medical oncologists, while they accounted for 500 in Egypt [6].

#### The lack of reliable electronic resources

This lack exacerbates the scarcity of cancer-related information, and translates to a much more needed effort to properly estimate some practicalities: the number of study centres to include; the average trial screening target; and the expected timeframe and budget.

#### Limited qualified staff trained on the management and monitoring of clinical trials

Only a few master’s degrees in clinical research training are accessible after a 4-year bachelor’s degree in a healthcare-related field. Most are awarded by private universities. Aside from that, in a country where medical research is still at a nascent stage, local investigators may have not yet attained a long-standing experience and the operational expertise required to manage clinical trials, of any given size and complexity. This gap between the willingness to take part in cutting-edge research and the lack of formal training is a major reason why local physicians may find research daunting.

#### The unpredictability of regulatory timelines

This translates into a long and winding road from the approval of the Ministry of Health, the Regional Committee on the Protection of People and the National Commission for the Protection of Personal Data to the local ethical review committees. The complexity of the administrative framework creates biases that derail future research in the country, and undermine physicians’ endeavours to draw the interest of pharmaceutical companies and attract advanced therapeutic solutions to their deserving patients.

#### Ethical validity

While parsing the set of reasons, the JCO review questioned the ability of African countries to meet the International Clinical Practice standards and ethical validity, particularly regarding the readability of informed consent forms by patients. The concern is that if patients cannot read or comprehend written materials, in the form of long complex paperwork that is provided to them, their autonomous decision of participating in the research cannot be guaranteed. The fact is that this barrier is not less controversial and arguable. The therapeutic misconception among research subjects is not particular to African countries. Joffre et al [7] conducted a cross-sectional survey to measure the quality of understanding among adult patients participating in clinical trials that assessed a cancer-directed treatment at Dana-Farber Cancer Institute, a world-class institution and its affiliated institutions. The results noted that although most patients easily understood their consent forms, 70% did not understand that the experimental treatment was unproven and 74% did not recognise that the treatment was not standard. This begs the question of whether consent forms are written in a manner that conforms to plain language standards and aim to transcend linguistic and cultural barriers for effective global access to clinical trials.

### Cancer research in Morocco: a bibliometric analysis

Generating country-specific evidence is the key element to implement health policies that can be tailored to the specific needs of each population. According to the public database of biomedical literature, PubMed, the number of cancer-related publications in Morocco has risen from 105 in 2010 to 344 in 2021. Of note, this increasing trend has been stronger than in other conditions. Bibliometric analysis suggests that Morocco is ranked among the top African countries in terms of the most indexed publications. Overall, North African countries showed the highest growth in the country. Egypt has the highest number of indexed documents, H-index and the most cited documents with 67% of the total publications from 2015 to 2019, according to the Web of Science database [8]. Most papers are published in local or low-ranking journals. The exorbitant processing fees by peer-reviewed journals, the lack of funding opportunities, the high cost of many interventions and the limited resources and infrastructures render high-quality research arduous to achieve.

### Local strategies adopted to improve hosting clinical trials

In countries with challenging health issues, small efforts can lead to consequent results. If carefully planned, plenty of room exists to not cut corners on the conduction of clinical trials in LMICs. In contrast to their week trial density, 85% of the top 20 countries ranked by highest annual growth rate—in the period 2006–2012—were countries with emerging economies. The highest average rate was reported in lower-middle-income countries (14.7%) compared to 6.1% in high-income countries [2]. This remarkable growth reflects increasing awareness and scientific commitment among LMICs in recent years and is expected to continue rising. The enormous research potential offered by LMIC is lying on the increased incidence of cancer and especially the markedly lower cost of running clinical trials. Cost-effectiveness drives the research agenda of pharmaceutical companies that seek the best value for money with a maximisation of profit.

Morocco was the first country in North Africa to initiate a National Cancer Prevention and Control Plan [9]. A real keystone of modern public health policy, the plan advocates concrete and long-standing operational measures to implement the four basic components of cancer control: prevention, early detection, diagnosis and treatment, and palliative care. The plan was tasked with assisting and promoting cancer research and made sustained progress across the entire research continuum.

The efforts were achieved with the foundation of the National Cancer Research Institute (CRI). The referral academic institution with autonomous funding was dedicated to leveraging the standards of clinical research capabilities in the country. Since its creation in 2014, CRI has funded more than 40 research projects and created a national network for researchers in oncology with a mission to facilitate collaboration and coordination of cancer research in Morocco.

Accordingly, the number of designated cancer facilities has doubled over the past decade: The national coverage was enhanced further by two university medical centres, four regional oncology centres and creation of proximity oncology centres in provincial hospitals, alongside private specialised clinics.

In parallel, regulatory efforts have been made to improve the legislative framework governing the conduct of clinical trials. Public health agencies and institutional research ethics boards are adapting clear-sighted policies to accelerate the regulatory approval pathway. This translates into reduced time to first site initiation recruitment of 3–4 months, rather than 2 years, as asserted by local investigators.

Last but not least, when referring to clinical trials holders’ expectations, typically the goodwill, advocacy and competence of local physicians to manage clinical trials, we believe that there is room for trust. The reasons behind this motivation are tended to be self-evident. With regard to the widening gap between high-income and middle- and low-income countries, the value and oversight of clinical research are more relevant in our settings and much needed to scale up our capacity to control cancer. Consequently, we are more aware of the pertinence of proposing our patients for enrollment in clinical trials, as it might be their only way to benefit from advanced treatments and better health outcomes. It can hence be reasonably hoped to grow the confidence on local healthcare professionals and their ability to perform at the required standard of advanced practice.

## Conclusion

Most clinicians practicing in the fields of cancer care in LMICs are willing to take part in clinical research, but a major structural barrier for their participation, especially in clinical trials, pertains to the availability of the trial in their work area. This single challenge affects the clinical workload and delivery of care among clinical oncologists and jeopardises the efforts to improve health equity.

## Conflicts of interest

The authors declare that they have no conflicts of interest.

## Funding

No funding was received for this manuscript.

## Figures and Tables

**Table 1.** Global overview of active clinical trials in Maghreb countries.

Clinical trial identifier	Trial design	Condition	Intervention	Sponsor Institution	Status	Country
**Impassion 132** NCT03371017	Interventional phase III, double blind, placebo control, randomised	Early recurrent, advanced triple negative breast cancer	Atezolizumab or placebo plus chemotherapy	Pharmaceutical company	Recruiting	Algeria Morocco
**SAFIA** NCT03447132	Interventional phase III, double blind, placebo control, randomised	Operable luminal breast cancer	Fulvestrant vs fulvestrant plus palbociclib	Pharmaceutical company + funding from research organisation	Active not Recruiting	Algeria Morocco Tunisia
**SKYSCRAPER-07-** NCT04543617	Interventional phase III, double blind, placebo control, randomised	Unresectable oesophageal squamous cell carcinoma	Tiragolumab or placebo plus atezolizumab	Pharmaceutical company	Pending approval from health authorities	Morocco
**ICRG0201** NCT05190094	Interventional, open label, phase II, single-arm trial	Hormonal-positive HER2-negative advanced breast cancer,	Combination of palbociclib and aromatase inhibitor. Prediction of treatment efficacy using liquid biopsies.	Research organisation	Recruiting	Algeria
